# Supplementary material for: Compositional Variations between Adult and Infant Skin Microbiome: An Update
Source: Microorganisms. 2023 Jun 2;11(6):1484. doi: 10.3390/microorganisms11061484 (PMC10304506; doi:10.3390/microorganisms11061484)
Supplement: Supplementary file 1 [file microorganisms-11-01484-s001.zip › Supplementary tables.pdf]

SUPPLEMENTARY TABLES

Supplementary Table S1: QIIME2 Software Parameters

**Software Parameter**	**Value**
FastQC filtering min gc percentage	25
FastQC filtering max gc percentage	75
FastQC filtering min average base quality	20
DADA2 forward read trim position	21
DADA2 reverse read trim position	19
DADA2 forward read truncation position	No truncation
DADA2 reverse read truncation position	No truncation
BLAST+ penalty	-5
BLAST+ reward	4
BLAST+ gapopen	5
BLAST+ gapextend	5

Supplementary Table S2: Software Versions

**Software**	**Version**
FastQC:	v0.11.5
MultiQC	1.7
QIIME2	2019.4
DADA2	QIIME2 2019.1
BLAST+	2.6.0+
scikit-learn	QIIME2 2019.1
Picrust2	2.3.0_b

Supplementary Table S3: Species level taxa utilised for network analysis

Taxon No.	Taxon Classification	Taxon No.	Taxon Classification	Taxon No.	Taxon Classification
1	*Abiotrophia defectiva*	46	*Gemella sanguinis*	91	*Rothia aeria*
2	*Acinetobacter baumannii*	47	*Granulicatella adiacens|paradiacens*	92	*Rothia mucilaginosa*
3	*Acinetobacter haemolyticus*	48	*Granulicatella elegans*	93	*Schaalia odontolytica*
4	*Acinetobacter radioresistens*	49	*Haemophilus parainfluenzae*	94	*Staphylococcus aureus*
5	*Acinetobacter A58*	50	*Haemophilus sp_908*	95	*Staphylococcus capitis*
6	*Acinetobacter DR1*	51	*Janthinobacterium sp_SY12*	96	*Staphylococcus epidermidis*
7	*Actinomyces graevenitzii*	52	*Kocuria rhizophila*	97	*Staphylococcus haemolyticus*
8	*Actinomyces sp_172*	53	*Kytococcus sedentarius*	98	*Staphylococcus hominis*
9	*Actinomyces sp_180*	54	*Lactobacillus crispatus*	99	*Staphylococcus pasteuri|warneri*
10	*Actinomyces sp_181*	55	*Lactobacillus iners*	100	*Staphylococcus saprophyticus*
11	*Actinomycetales sp_C05*	56	*Lactobacillus jensenii*	101	*Staphylococcus saprophyticus*
12	*Aeromonas hydrophila*	57	*Lactococcus lactis*	102	*Streptococcus anginosus*
13	*Alloprevotella sp_473*	58	*Lautropia mirabilis*	103	*Streptococcus australis*
14	*Anaerococcus hydrogenalis*	59	*Leptotrichia sp_215*	104	*Streptococcus dentisani|mitis*
15	*Anaerococcus octavius*	60	*Macrococcus caseolyticus*	105	*Streptococcus dentisani|mitis|peroris*
16	*Atopobium parvulum*	61	*Micrococcus luteus*	106	*Streptococcus gordonii*
17	*Atopobium vaginae*	62	*Micrococcus luteus_Oral_Taxon_C78*	107	*Streptococcus infantis*
18	*Bergeyella sp_H70*	63	*Micrococcus luteus_Oral_Taxon_H68*	108	*Streptococcus lactarius*
19	*Brachybacterium sp_D23*	64	*Micrococcus sp._Oral_Taxon_B64*	109	*Streptococcus mitis*
20	*Brevundimonas diminuta*	65	*Micrococcus sp._Oral_Taxon_F54*	110	*Streptococcus mitis sp_C300*
21	*Campylobacter ureolyticus*	66	*Moraxella osloensis*	111	*Streptococcus mitis sp_M334*
22	*Capnocytophaga leadbetteri*	67	*Neisseria cinerea|meningitidis*	112	*Streptococcus oralis*
23	*Capnocytophaga sputigena*	68	*Neisseria elongata*	113	*Streptococcus parasanguinis_II*
24	*Corynebacterium accolens*	69	*Neisseria flavescens|subflava*	114	*Streptococcus parasanguinis*
25	*Corynebacterium amycolatum*	70	*Neisseria mucosa*	115	*Streptococcus peroris*

26	*Corynebacterium aurimucosum*	*71*	*Pantoea ananatis*	*116*	*Streptococcus salivarius*
27	*Corynebacterium genitalium*	*72*	*Peptoniphilus harei*	*117*	*Streptococcus sanguinis*
28	*Corynebacterium jeikeium*	*73*	*Peptoniphilus lacrimalis sp_836*	*118*	*Streptococcus sp_061*
29	*Corynebacterium kroppenstedtii*	*74*	*Peptoniphilus sp_A87*	*119*	*Streptococcus sp_064*
30	*Corynebacterium lipophiloflavum*	*75*	*Peptostreptococcus anaerobius*	*120*	*Streptococcus sp_074*
31	*Corynebacterium mucifaciens*	*76*	*Porphyromonas pasteri*	*121*	*Streptococcus sp_423*
32	*Corynebacterium pseudogenitalium*	*77*	*Porphyromonas sp_930*	*122*	*Streptococcus sp_C150*
33	*Corynebacterium sp_B71*	*78*	*Prevotella bivia*	*123*	*Streptococcus sp_C300*
34	*Corynebacterium sundsvallense*	*79*	*Prevotella corporis*	*124*	*Streptococcus thermophilus*
35	*Corynebacterium tuscaniae|tuscaniense*	*80*	*Prevotella disiens*	*125*	*unresolved genus*
36	*Cutibacterium acnes*	*81*	*Prevotella histicola*	*126*	*unresolved genus*
37	*Cutibacterium granulosum*	*82*	*Prevotella melaninogenica*	*127*	*Veillonella atypica*
38	*Dermacoccus sp_Ellin185*	*83*	*Prevotella nanceiensis*	*128*	*Veillonella denticariosi|dispar|parvula*
39	*Dialister micraerophilus*	*84*	*Prevotella timonensis*	*129*	*Veillonella dispar|parvula*
40	*Dietzia cinnamea*	*85*	*Propionibacterium sp_193*	*130*	*Veillonella sp_780*
41	*Enhydrobacter aerosaccus*	*86*	*Pseudomonas antarctica*		
42	*Finegoldia magna*	*87*	*Pseudomonas gingeri*		
43	*Fusobacterium nucleatum*	*88*	*Pseudomonas putida*		
44	*Fusobacterium periodonticum*	*89*	*Psychrobacter sp_cryopeg55*		
45	*Gemella haemolysans*	*90*	*Roseomonas mucosa*		
