# Supplementary figures and images for: Compositional Variations between Adult and Infant Skin Microbiome: An Update
Source: Microorganisms. 2023 Jun 2;11(6):1484. doi: 10.3390/microorganisms11061484 (PMC10304506; doi:10.3390/microorganisms11061484)

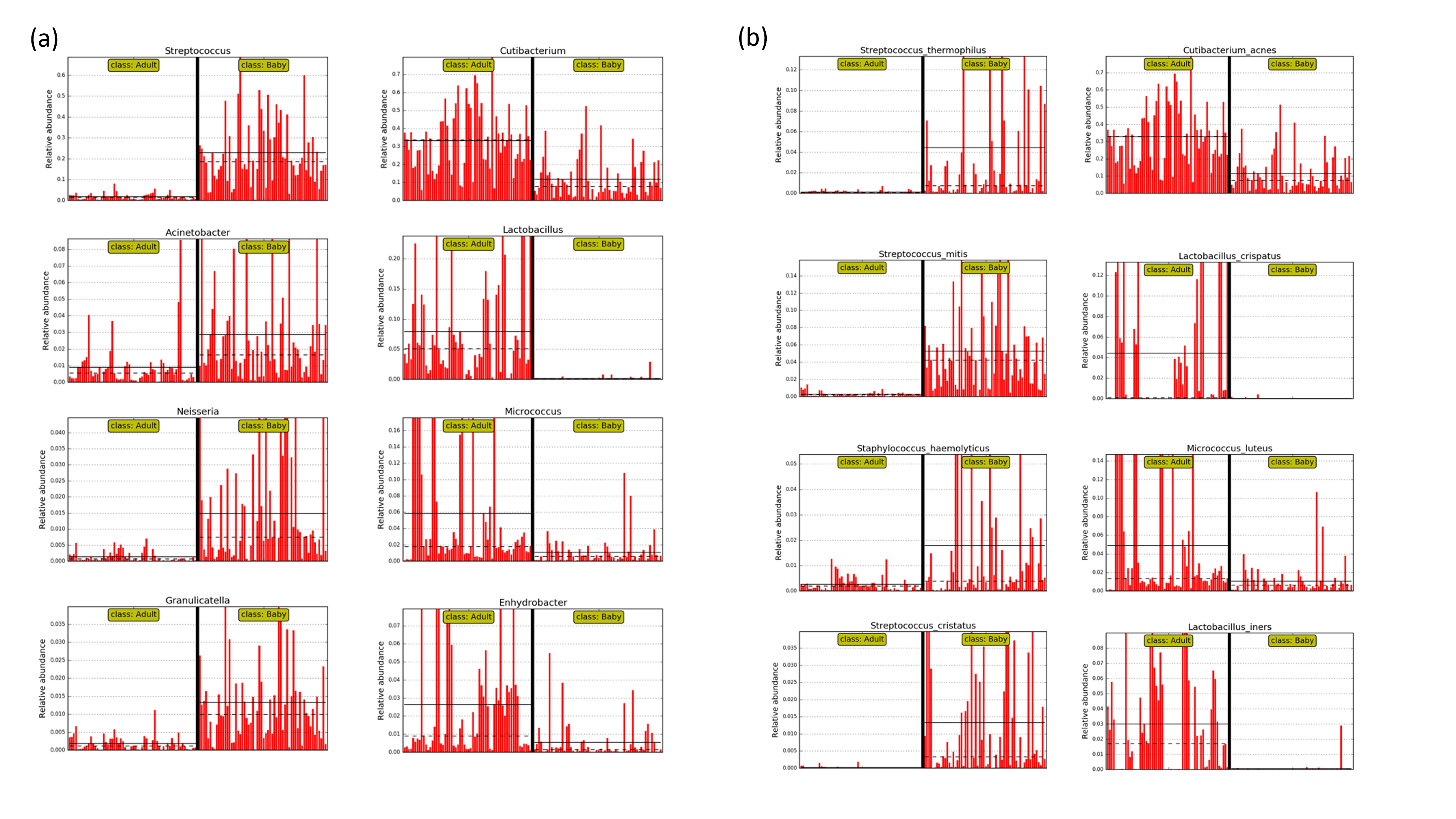

Supplement: Supplementary file 1 [file microorganisms-11-01484-s001.zip › Supp Figure S1.tif]
